# Supplementary figures and images for: Characterising antibody kinetics from multiple influenza infection and vaccination events in ferrets
Source: PLoS Comput Biol. 2019 Aug 19;15(8):e1007294. doi: 10.1371/journal.pcbi.1007294 (PMC6715255; doi:10.1371/journal.pcbi.1007294)

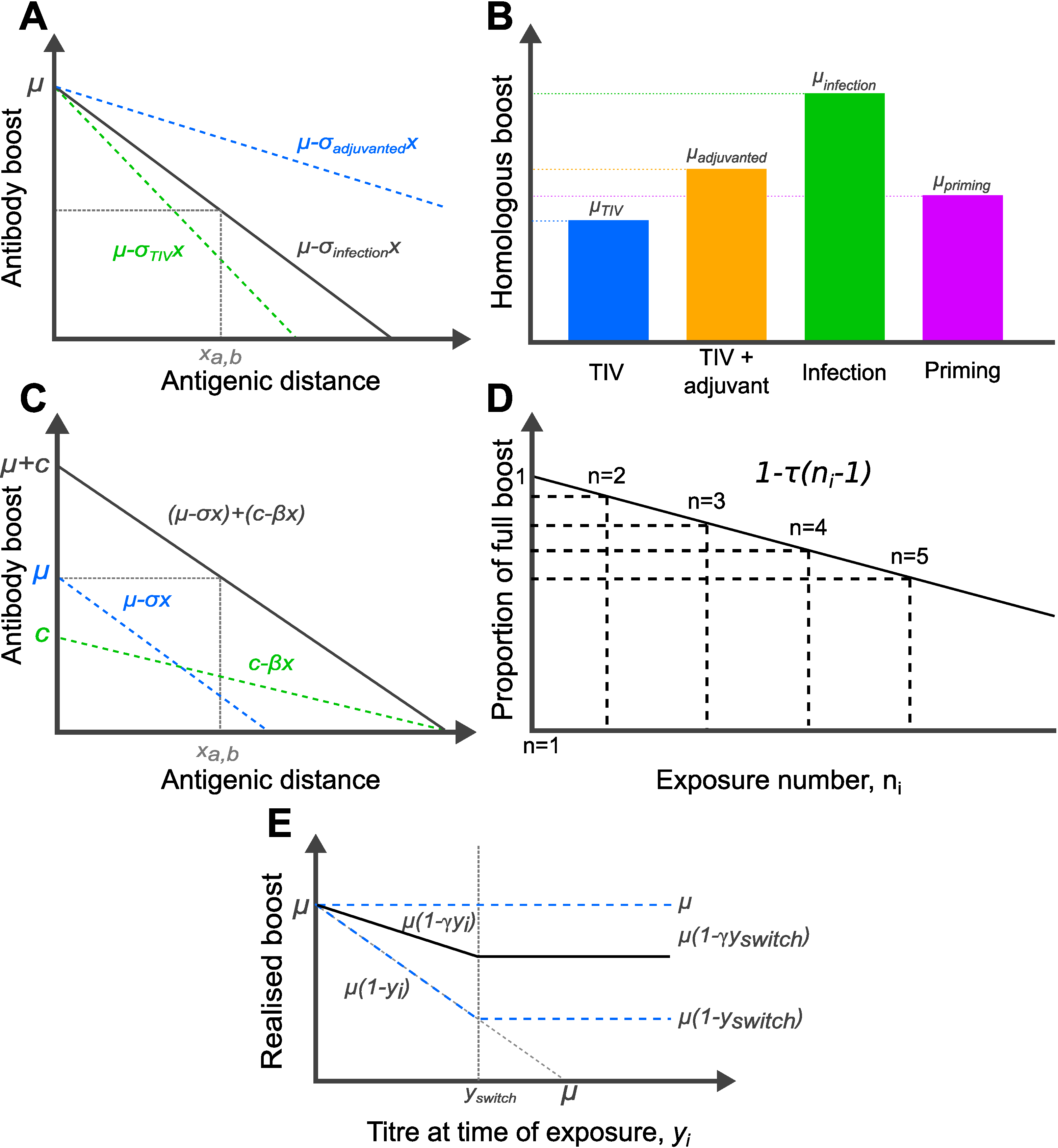

Supplement: S1 Fig — A: Cross reactive antibody boosting. The degree of boosting decreases as the antigenic distance between the exposure and measured strain increases. Different exposure types may have different gradients; B: Illustrative example of exposure type specific parameter values. Level of homologous boosting may depend on the exposure type. Note that this may also apply to other parameters eg. waning rate; C: Joint effect of exposure boosting and priming infection. Full boosting following a primed exposure is the sum of contributions of the exposure itself and the effect of priming; D: Antigenic seniority mechanism. Amount of antibody boosting decreases linearly with the number of prior exposures; E: Titre dependent boosting. Solid black line shows example where 0 ≤ γ ≤ 1. Blue dashed lines show boundary conditions. Note that the realised boost does not change when yi is above yswitch. (TIF) [file pcbi.1007294.s008.tif]

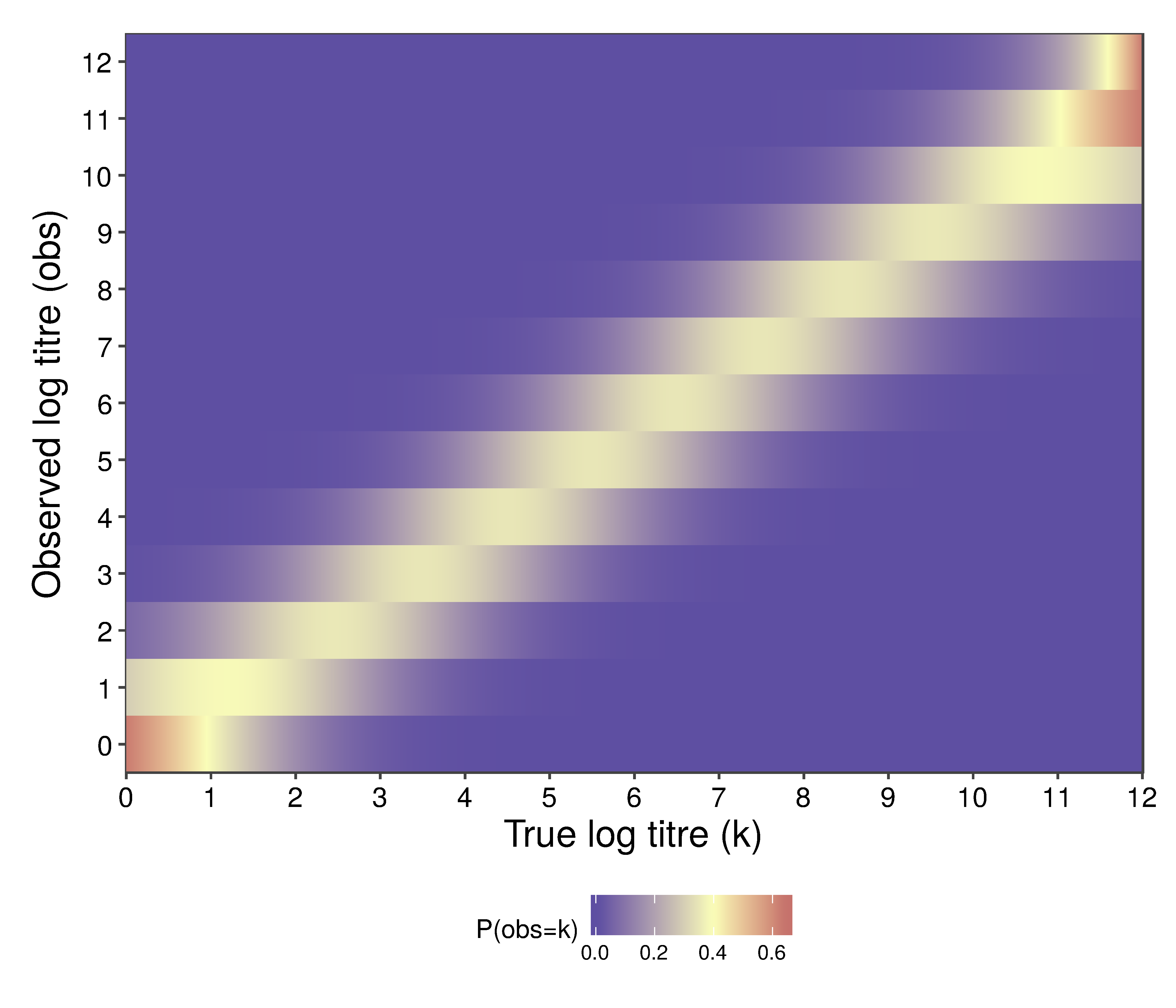

Supplement: S2 Fig — Probability of observing a particular log titre given an underlying true, latent titre. Note that the true titre is a continuous value, whereas observations are discrete. Furthermore, truncation of the distribution at the upper and lower limit of the assay results in an asymmetrical distribution when the true value is at either of these limits. True values outside of these limits will be observed as a value within the assay limits. (TIF) [file pcbi.1007294.s009.tif]

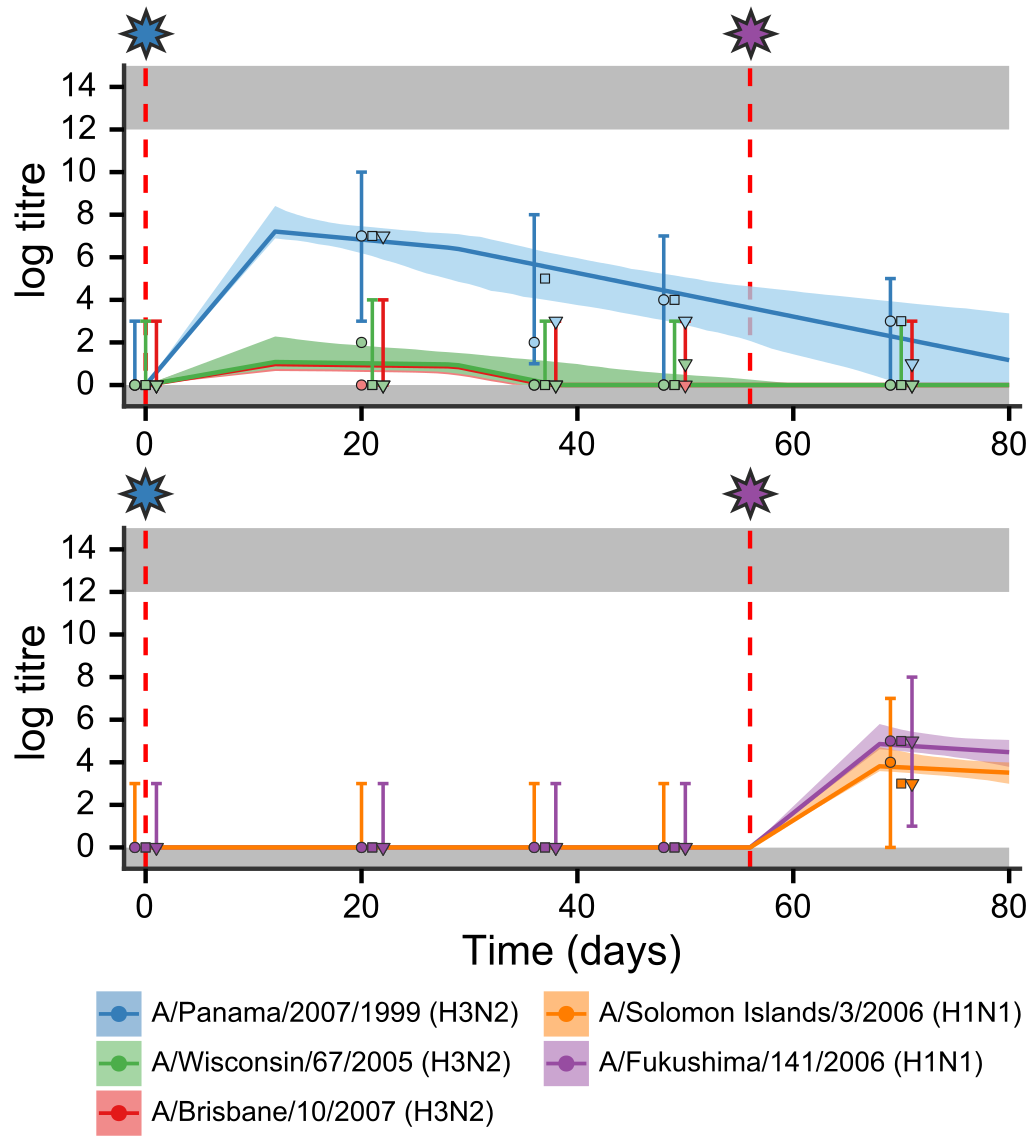

Supplement: S3 Fig — Equivalent to Fig 4E, but using a model assuming shared kinetics parameters between post A/H3N2 and A/H1N1 infection, between both adjuvanted TIVs and between both unadjuvanted TIVs. Solid coloured lines and shaded regions show posterior median and 95% credible intervals of latent titres. Points show observed antibody titres. Bars show 95% prediction intervals on observable titres. Red dashed lines show time of infection with A/Panama/2007/99 (H3N2) and A/Fukushima/141/2006 (H1N1) respectively. (TIF) [file pcbi.1007294.s010.tif]

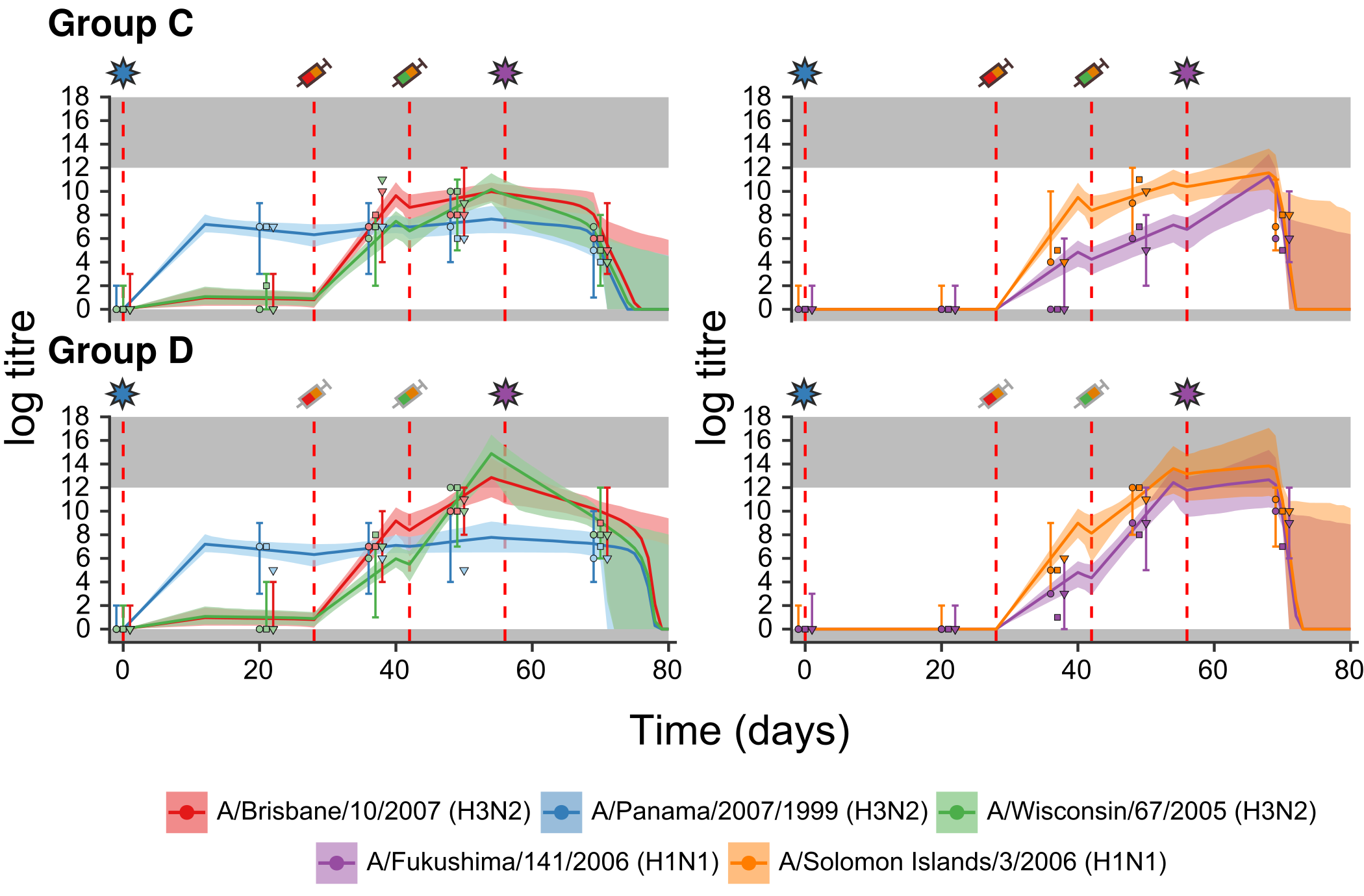

Supplement: S4 Fig — Equivalent to Fig 4C & 4D, but using a model with titre-dependent boosting. Solid coloured lines and shaded regions show posterior median and 95% credible intervals of latent titres. Points show observed antibody titres. Bars show 95% prediction intervals on observable titres. Red dashed lines show exposures as in Fig 1. (TIF) [file pcbi.1007294.s011.tif]

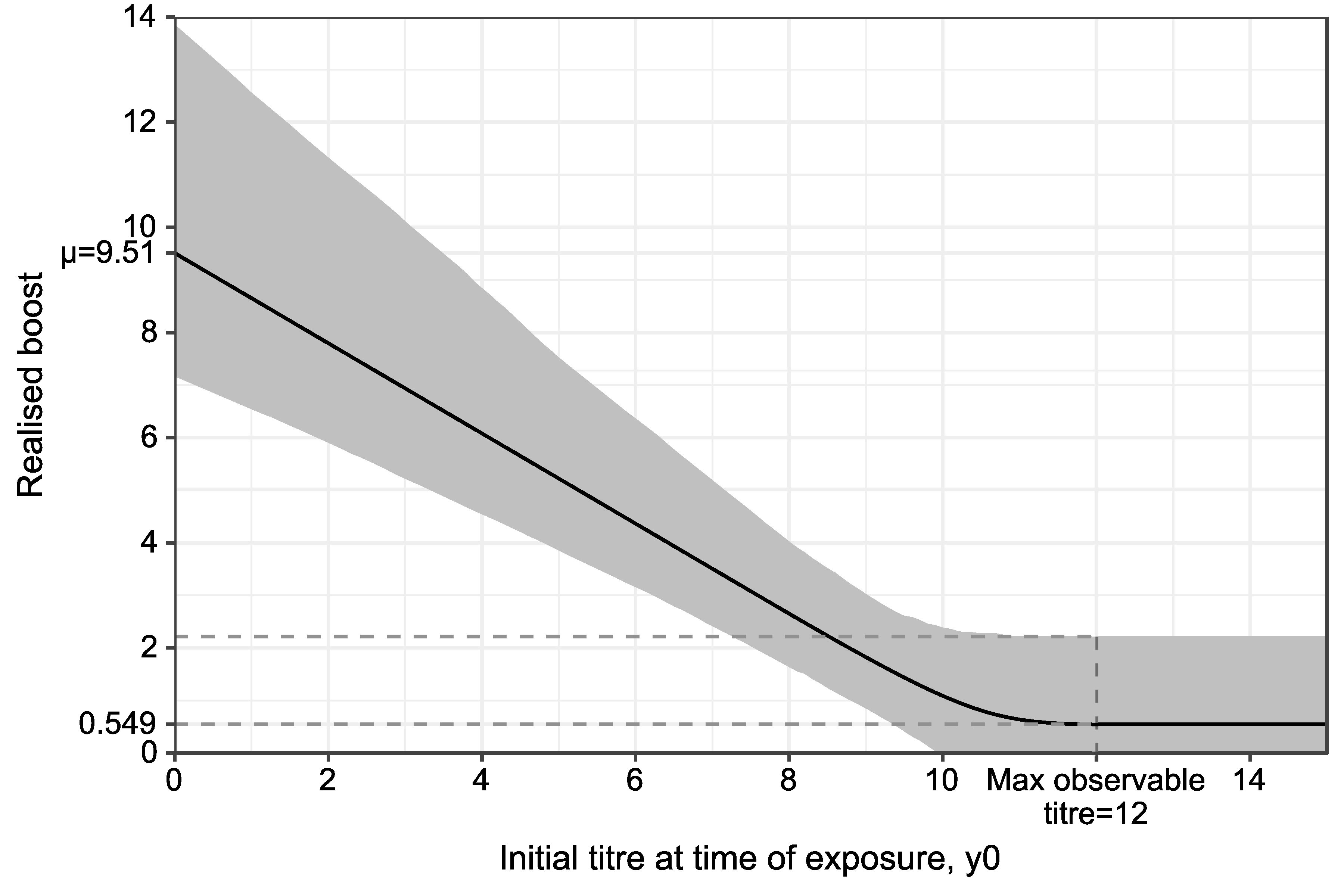

Supplement: S5 Fig — Shaded gray regions shows 95% credible intervals (CI) drawn from the multivariate posterior. Solid black line shows multivariate posterior mean; Dashed gray lines show median and 95% CI for realised antibody boosting from a titre of 12. (TIF) [file pcbi.1007294.s012.tif]

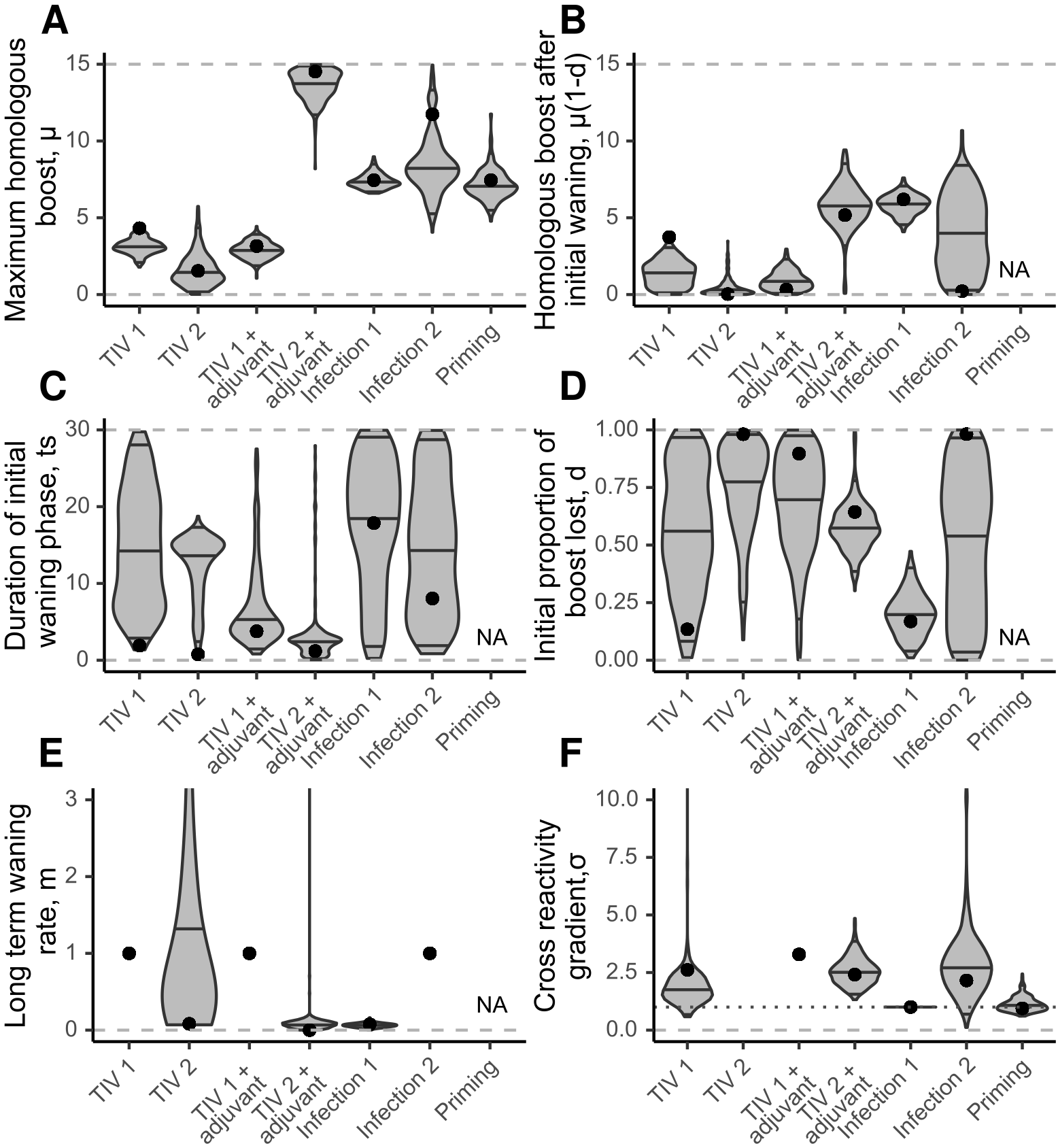

Supplement: S6 Fig — Violin plots show estimated posterior densities with medians and 95% credible intervals marked as horizontal black lines. Dashed gray lines show bounds on uniform prior. Black dots show true values. (A) Estimates for homologous boosting parameter, μ. (B) Estimates for homologous boost at the end of the initial waning period, μ(1 − d). (C) Estimates for duration of initial waning phase, ts. (D) Estimates for proportion of initial boost lost during the initial waning phase, d. (E) Estimates for long term waning rate, m. Estimates for TIV 1, TIV 1 + adjuvant and Infection 2 excluded due to lack of identifiability. (F) Estimates for cross reactivity gradient, σ. Note that this value is fixed at 1 for priming infection (Infection 1), shown by the horizontal dotted line. Values for TIV 2 and TIV 1 + adjuvant excluded due to lack of identifiability. (TIF) [file pcbi.1007294.s013.tif]

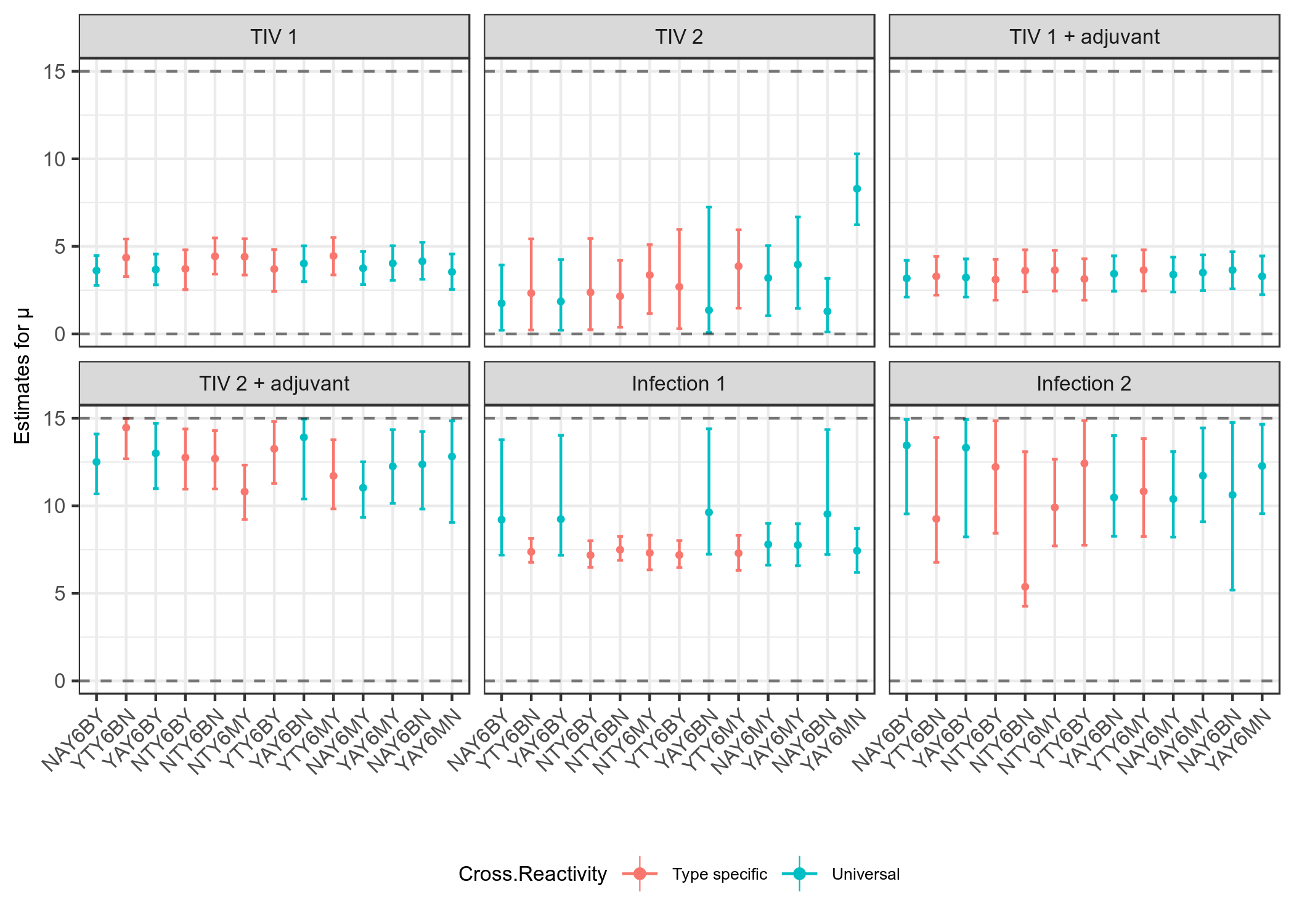

Supplement: S7 Fig — Points show posterior median; line ranges show 95% credible intervals. Estimates are stratified by exposure type and ordered in order of increasing ELPD. Estimates are coloured according to whether or not cross reactivity was assumed to be a universal parameter or type-specific. Dashed horizontal lines represent uniform prior range. Model codes on x-axis relate to the first letter of each mechanism as described in S2 Table. (TIF) [file pcbi.1007294.s014.tif]

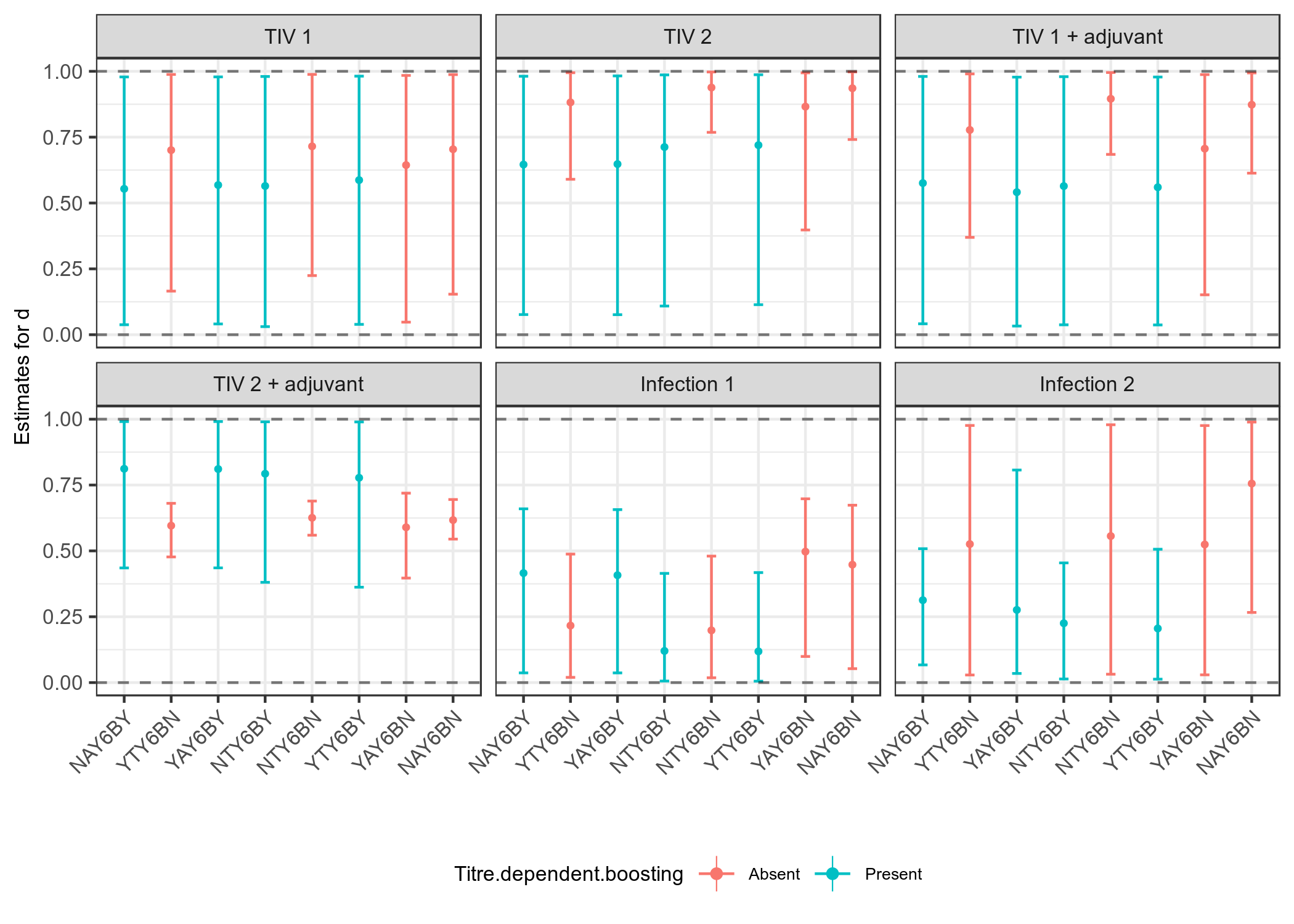

Supplement: S8 Fig — Points show posterior median; line ranges show 95% credible intervals. Estimates are stratified by exposure type and ordered in order of increasing ELPD. Estimates are coloured according to whether or not titre-dependent boosting was included. Dashed horizontal lines represent uniform prior range. Model codes on x-axis relate to the first letter of each mechanism as described in S2 Table. (TIF) [file pcbi.1007294.s015.tif]

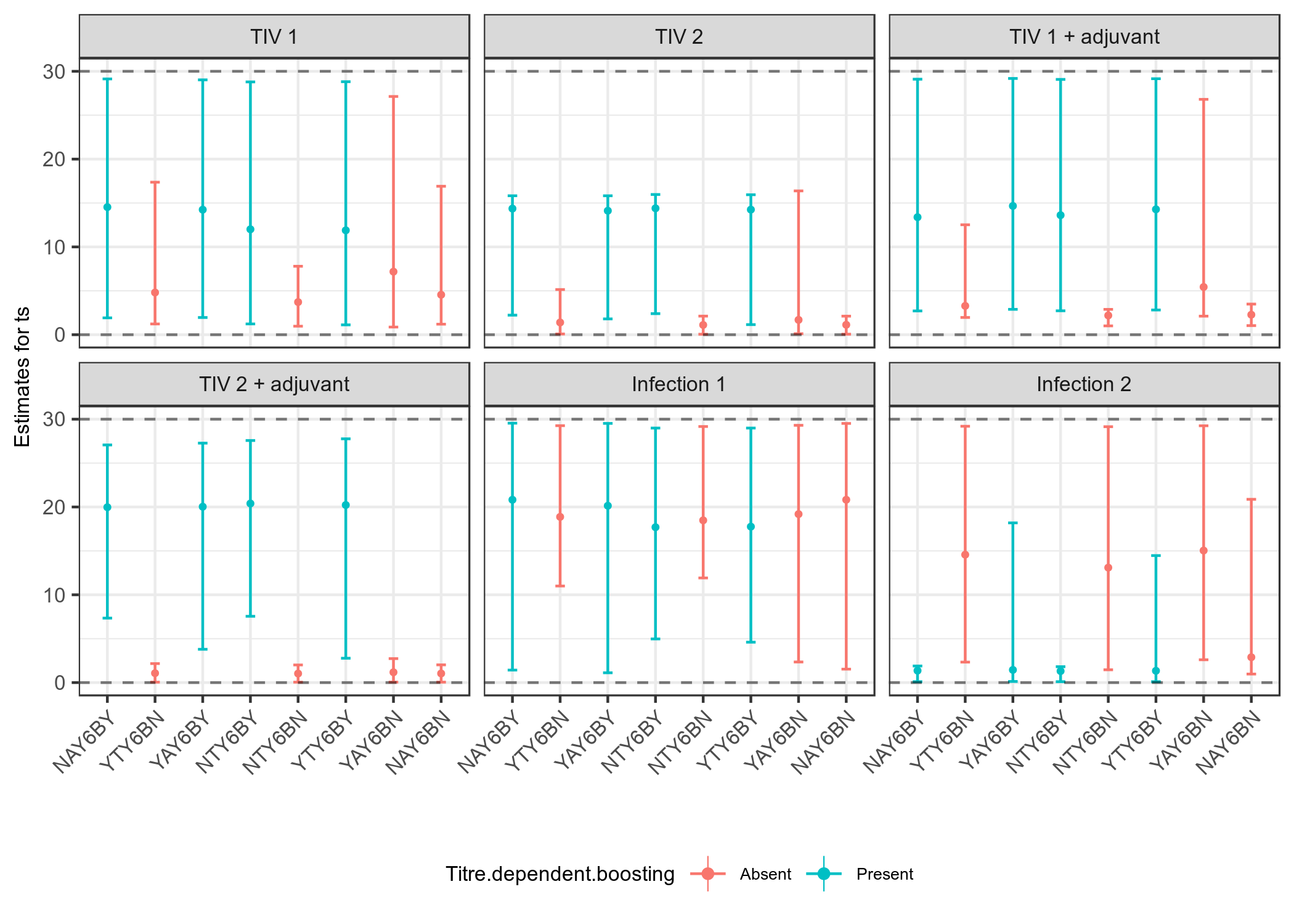

Supplement: S9 Fig — Points show posterior median; line ranges show 95% credible intervals. Estimates are stratified by exposure type and ordered in order of increasing ELPD. Estimates are coloured according to whether or not titre-dependent boosting was included. Dashed horizontal lines represent uniform prior range. Model codes on x-axis relate to the first letter of each mechanism as described in S2 Table. (TIF) [file pcbi.1007294.s016.tif]

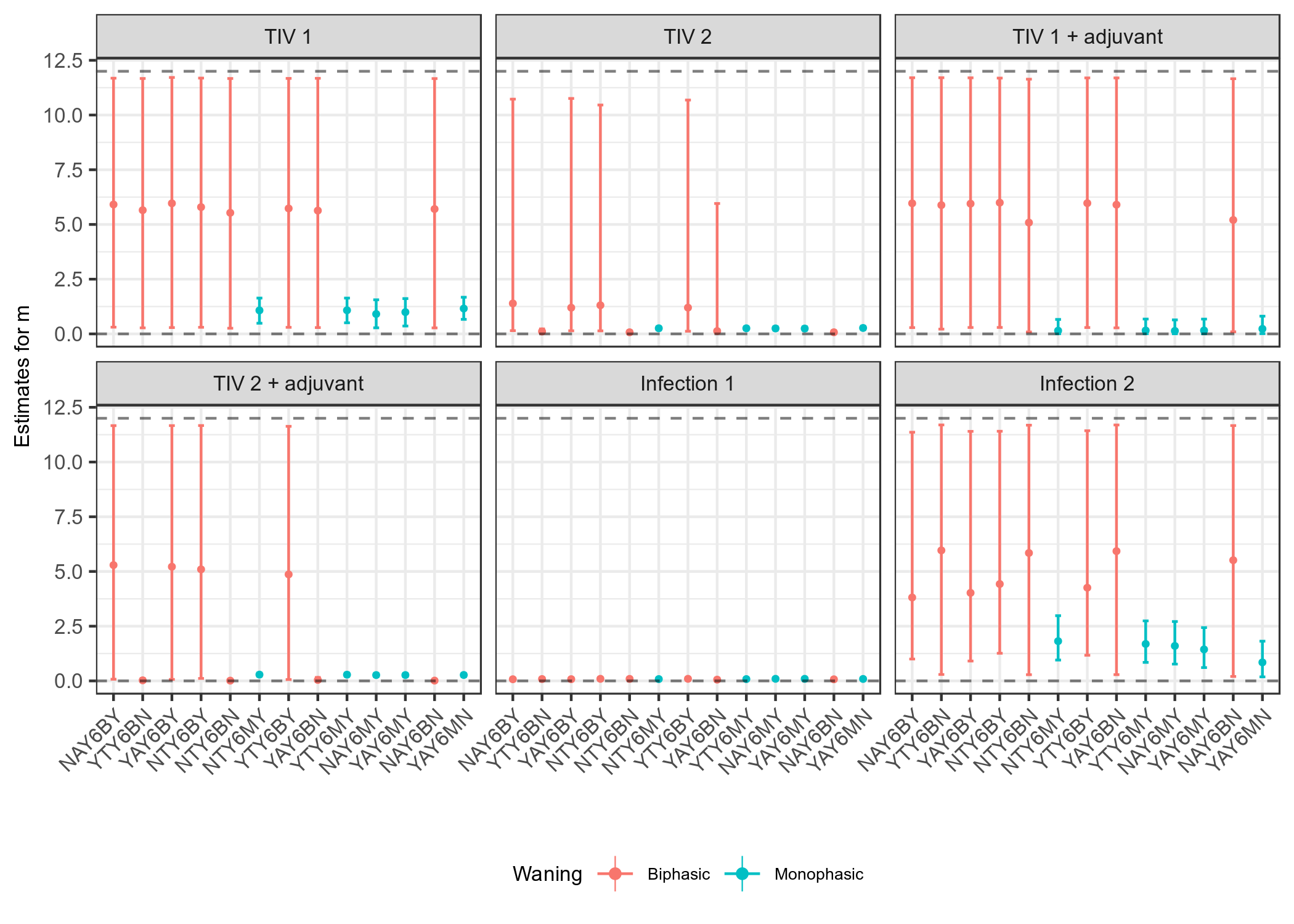

Supplement: S10 Fig — Points show posterior median; line ranges show 95% credible intervals. Estimates are stratified by exposure type and ordered in order of increasing ELPD. Estimates are coloured according to whether or not waning was assumed to be biphasic or monophasic. Dashed horizontal lines represent uniform prior range. Model codes on x-axis relate to the first letter of each mechanism as described in S2 Table. (TIF) [file pcbi.1007294.s017.tif]

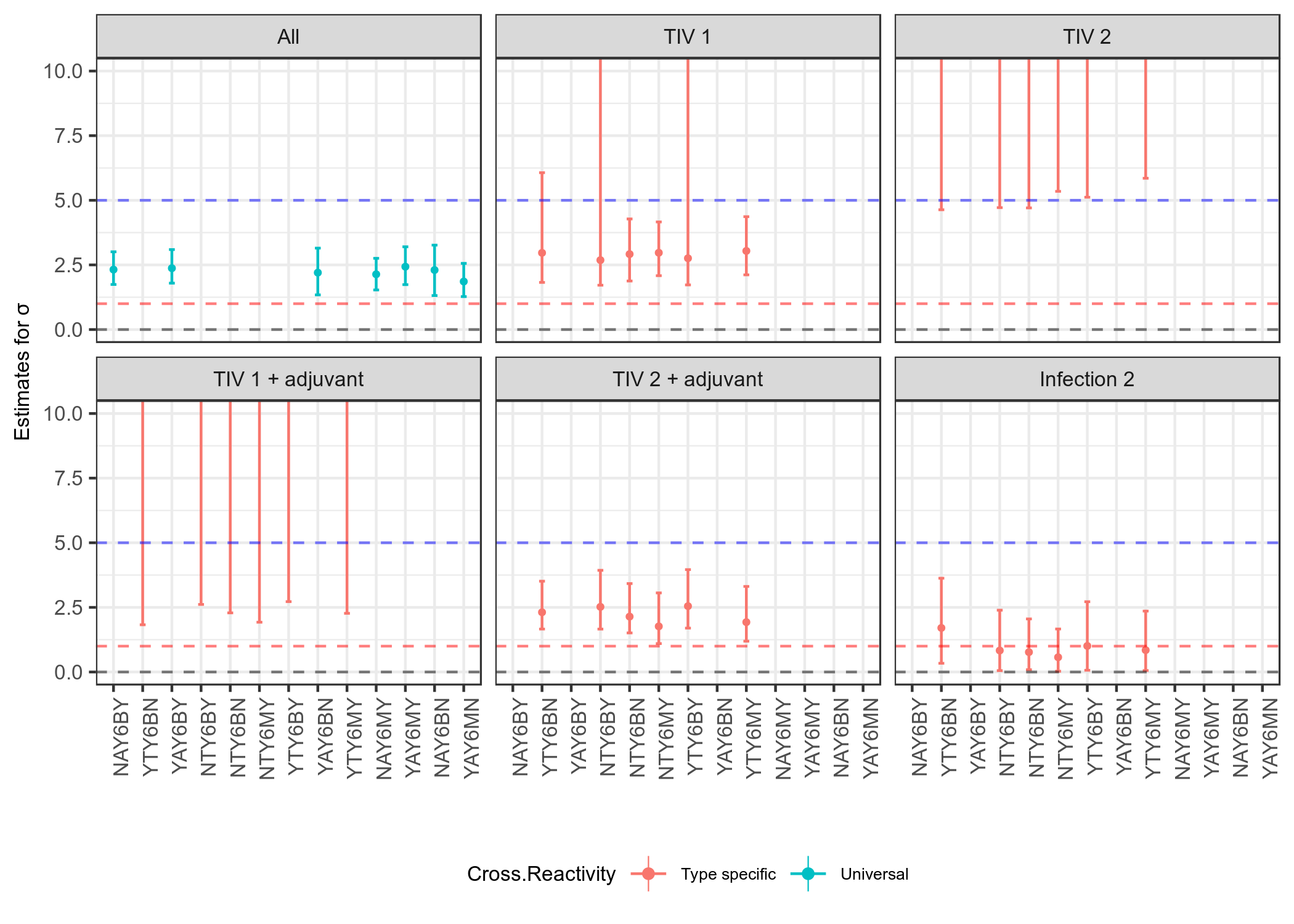

Supplement: S11 Fig — Points show posterior median; line ranges show 95% credible intervals. Estimates are stratified by exposure type and ordered in order of increasing WAIC. Estimates are coloured according to whether or not cross reactivity was assumed to be a universal parameter or type-specific. Plots are truncated from above at 10 for clarity, but upper prior bound was 100. Red dashed line shows the fixed value of σ = 1 for priming infection. Blue dashed line shows value above which a homologous boost of μ = 5 would give an observed boost of 0 against a strain with an antigenic distance of 1. Model codes on x-axis relate to the first letter of each mechanism as described in S2 Table. (TIF) [file pcbi.1007294.s018.tif]

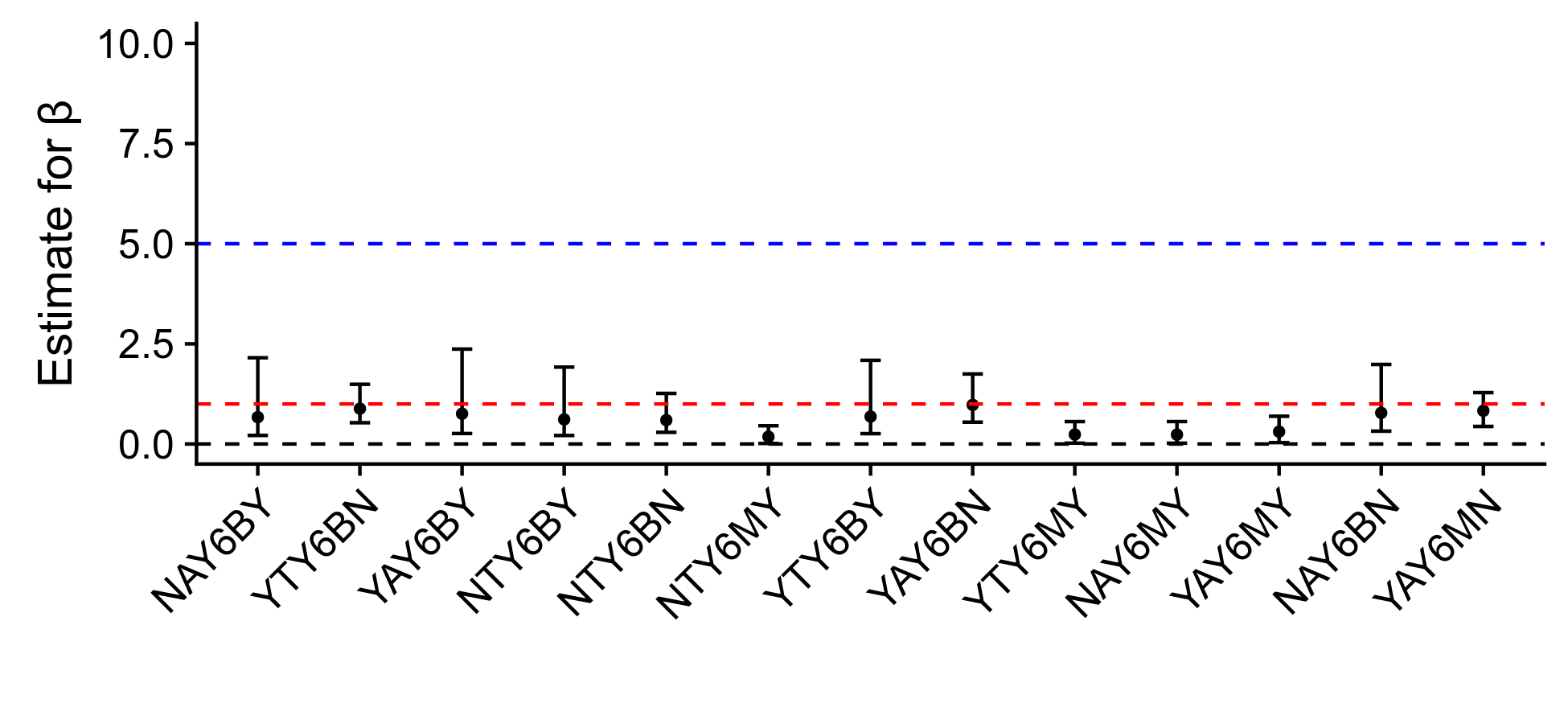

Supplement: S12 Fig — Points show posterior median; line ranges show 95% credible intervals. Red dashed line shows the fixed value of σ = 1 for priming infection. Blue dashed line shows value above which a homologous boost of μ = 5 would give an observed boost of 0 against a strain with an antigenic distance of 1. Estimates are ordered by increasing ELPD. Model codes on x-axis relate to the first letter of each mechanism as described in S2 Table. (TIF) [file pcbi.1007294.s019.tif]

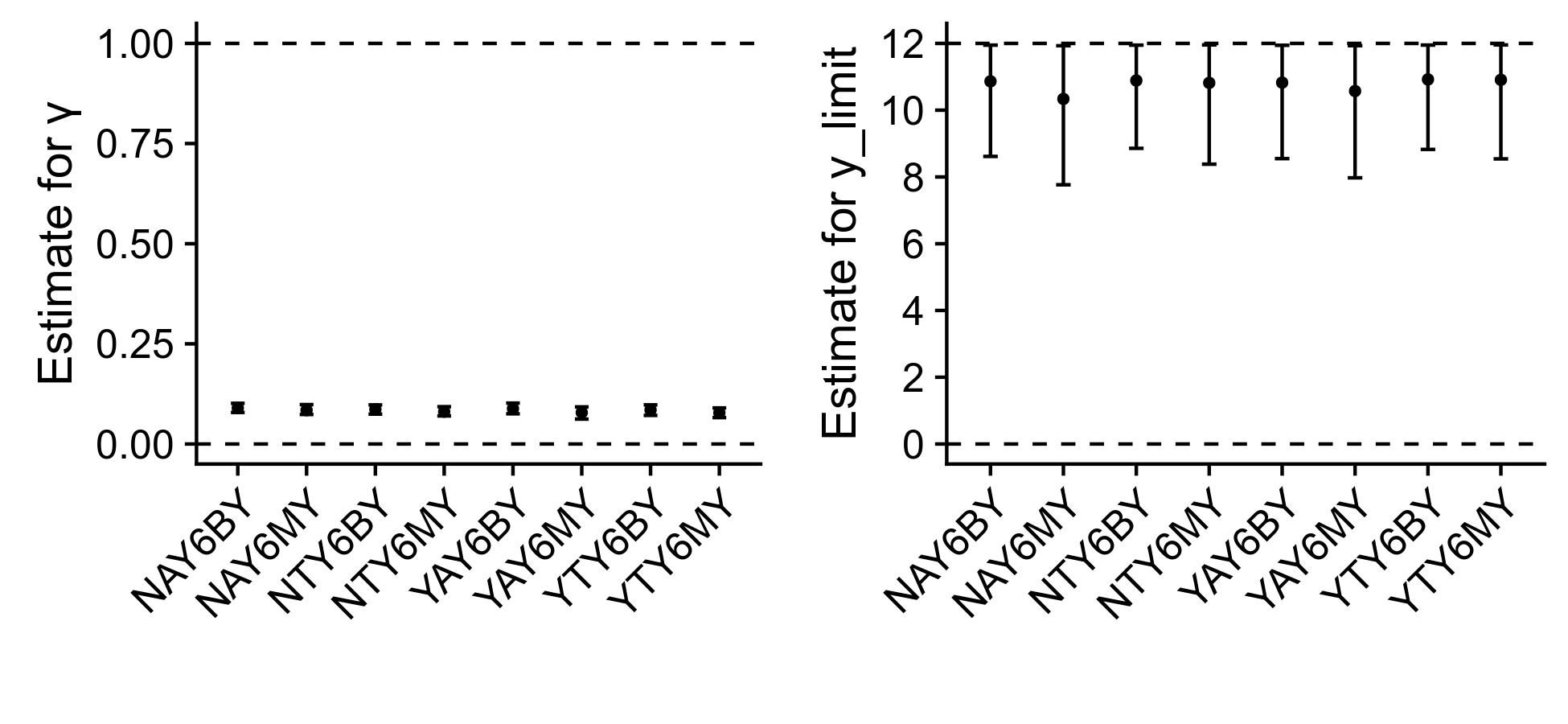

Supplement: S13 Fig — Points show posterior median; line ranges show 95% credible intervals. Estimates are ordered by increasing ELPD. Model codes on x-axis relate to the first letter of each mechanism as described in S2 Table. (TIF) [file pcbi.1007294.s020.tif]

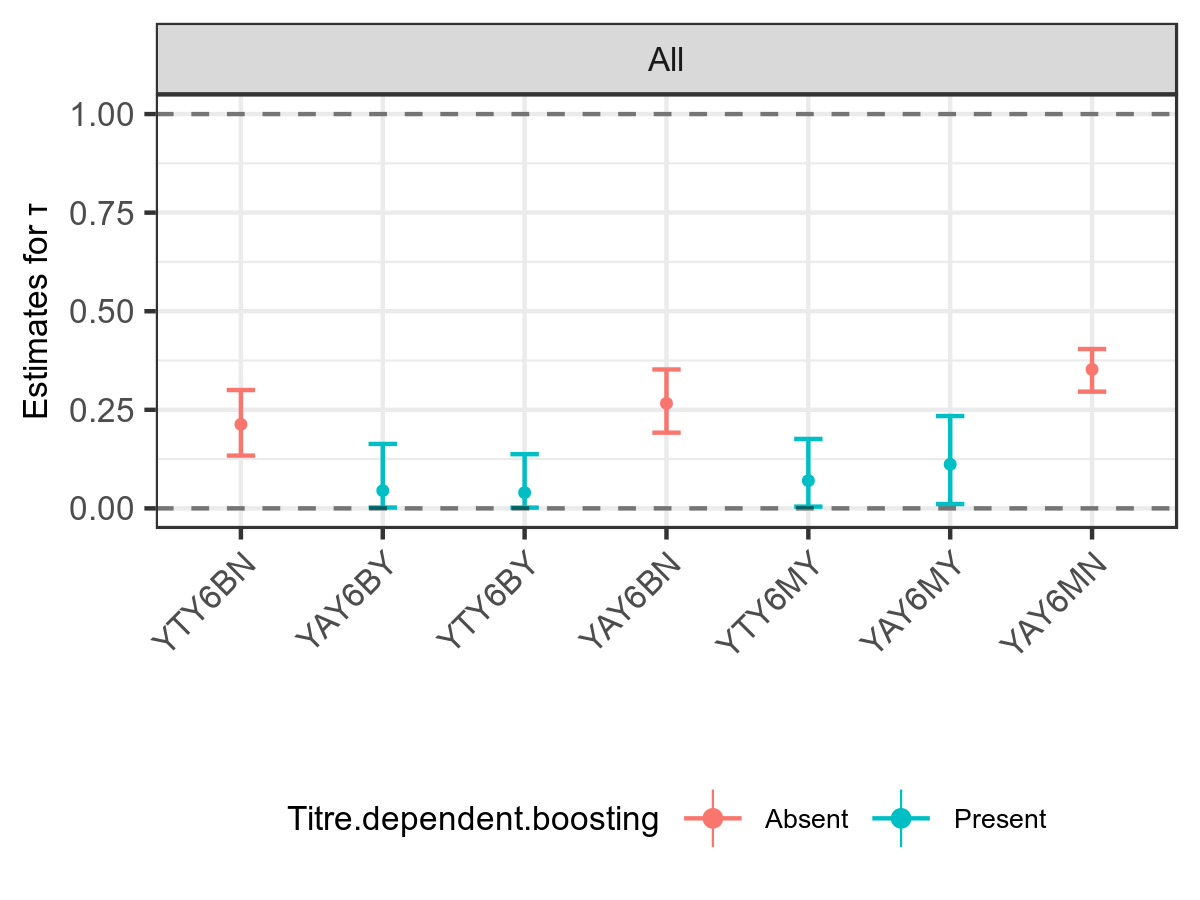

Supplement: S14 Fig — Points show posterior median; line ranges show 95% credible intervals. Estimates are ordered by increasing ELPD. Estimates are coloured according to whether or not titre-dependent boosting was also included in the model. Model codes on x-axis relate to the first letter of each mechanism as described in S2 Table. (TIF) [file pcbi.1007294.s021.tif]
